# Correction: Bowers et al. Tart Cherry Extract and Omega Fatty Acids Reduce Behavioral Deficits and Gliosis in the 5xFAD Mouse Model of Alzheimer’s Disease. *Brain Sci.* 2021, *11*, 1423

**DOI:** 10.3390/brainsci13060895

**Published:** 2023-06-01

**Authors:** Zackary Bowers, Panchanan Maiti, Ali Bourcier, Jarod Morse, Kenneth Jenrow, Julien Rossignol, Gary L. Dunbar

**Affiliations:** 1 Field Neurosciences Institute Laboratory for Restorative Neurology, Central Michigan University, Mt. Pleasant, MI 58859, USA; 2 Program in Neuroscience, Central Michigan University, Mt. Pleasant, MI 48859, USA; 3 College of Health and Human Services, Saginaw Valley State University, University Center, Saginaw, MI 48710, USA; 4 Department of Psychology, Central Michigan University, Mt. Pleasant, MI 48859, USA; 5 Field Neuroscience Institute Laboratory of Restorative Neurology, Ascension St. Mary’s Hospital, Saginaw, MI 48604, USA; 6 College of Medicine, Central Michigan University, Mt. Pleasant, MI 48859, USA

## 1. Incorrect Title

There is an error in the title of the published paper [1]. The correct title of the article should be **Tart Cherry Extract and Omega Fatty Acids Reduce Behavioral Deficits and Gliosis in the 5xFAD Mouse Model of Alzheimer’s Disease**. Our re-analyses of the histology indicated that the ameliorative effects of the treatment were primarily due to reduction in gliosis and that the trends toward decreasing pyknotic cells and amyloid-beta deposits were not significant. We apologize for this error and state that our major finding and all other scientific conclusions were unaffected. The original article has been updated.

## 2. Error in Figures

### 2.1. Figure 9

In the original article, there was a duplicate image for the 6-month 5xFAD in Figure 9A and the Figure 9B–D erroneously indicated significantly more pyknotic cells in the original publication. We removed incorrect images and corrected graphs.

In the original article, there was a mistake in the legend for Figure 9, which stated: “TBR treatment decreased pyknotic and tangle-like cells in the PFC and hippocampus of 5xFAD mice. (**A**) The 6- and 12-month-old 5xFAD and age-matched control mice were treated with (60 mg/kg BW) or vehicle for 2 months. (**B**) There was a significant difference in the number of pyknotic cells for 6- and 12-month-old mice in the PFC. (**C**) There was a significant difference in the number of pyknotic cells for 6- and 12-month-old mice in the CA1 area of the hippocampus. (**D**) In the CA3 area of the hippocampus there was a significant difference in the number of pyknotic cells for the 6-month-old animals but not for the 12-month-old mice. * *p* < 0.05.” However, our re-analyses indicated no differences in the cresyl-violet counts for PFC, CA1, or CA3. The correct legend appears below. 

**Figure 9:**
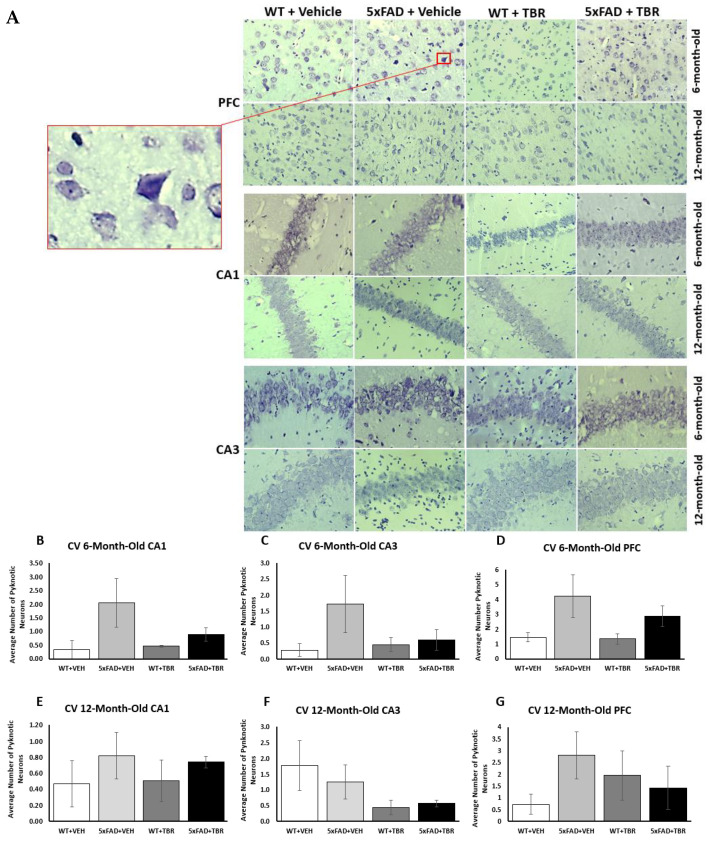
Cresyl-violet stained tissue revealed pyknotic cells (see insert) in the pre-frontal cortex (PFC) and the CA1 and CA3 regions of the hippocampus in all mice (**A**). There was a non-significant trend toward more pyknotic cells in the 5xFAD mice in all but the CA3 area of the 12-month mice and treatments of TBR had no significant effects (**B**–**G**).

### 2.2. Figure 10

In the original article, there was a mistake in Figure 10 as published. We removed original graphs and inserted corrected graphs. The corrected Figure 10 appears below. Upon reanalysis, it was found that there was not a significant difference in the average amount of Aβ deposition. 

In the original article, there was a mistake in the legend for Figure 10, which stated: “Effect of TBR on Aβ plaque deposits in 5xFAD mice. (**A**) Representative image from three locations: the dentate gyrus (DG), retrosplenial cortex (RSC), and the entorhinal cortex (EC) for 6- and 12-month-old animals. (**B**) Using a two-tailed *t*-test, there was a significant difference in total amyloid β deposits in the retrosplenial cortex between 5xFAD 6-month-old mice treated with TBR and 5xFAD mice treated with vehicle. (**C**) Using a one-tailed *t*-test, there was a significant difference in total amyloid β deposits between 5xFAD 12-month-old mice treated with TBR and 5xFAD mice treated with vehicle. * *p* < 0.05.” The correct legend appears below. Upon reanalysis, it was found that there was not a significant difference in the average amount of Aβ deposition. 

**Figure 10:**
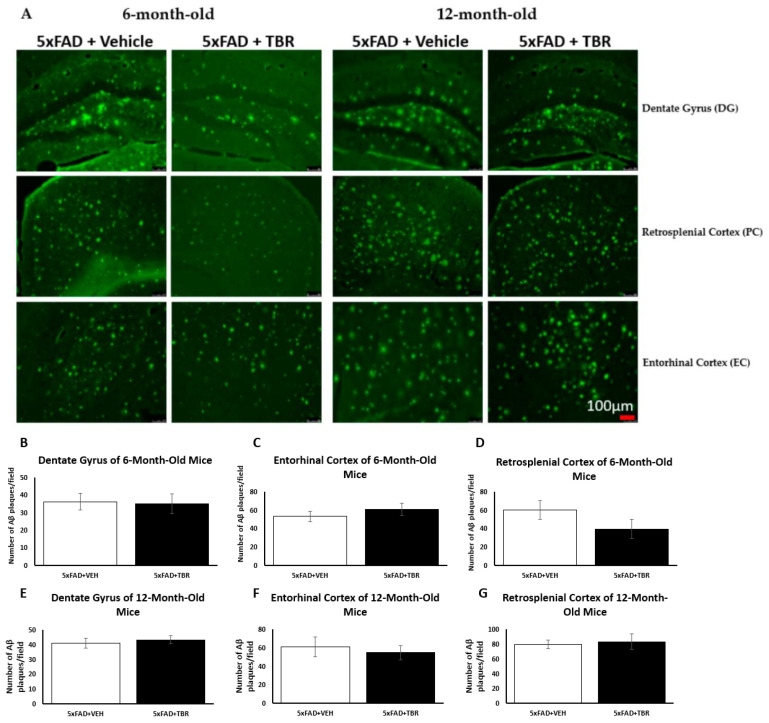
Effect of TBR on Aβ plaque deposits in 5xFAD mice. (**A**) Representative image from three locations: the dentate gyrus (DG), retrosplenial cortex (RSC), and the entorhinal cortex (EC) for 6- and 12-month-old animals. No significant differences were found between the 5xFAD mice and the 5xFAD mice treated with TBR for 6-or 12-month-old mice (**B**–**G**).

### 2.3. Figure 11

In the original article, there was a mistake in Figure 11 as published. We removed original graphs and inserted corrected graphs. The corrected Figure 11 appears below. Upon reanalysis, it was found that there was not a significant difference between male and female mice for the average amount of Aβ deposition. 

In the original article, there was a mistake in the legend for Figure 11, which stated: “TBR reduced the number of amyloid β deposits in male 5xFAD mice. The 6-month-old male 5xFAD mice treated with TBR had significantly less amyloid β in the retrosplenial cortex (RSC) than did male 5xFAD mice treated with vehicle and female AD mice treated with TBR or vehicle (**A**). The 6-month-old male 5xFAD mice treated with TBR had significantly less amyloid β in the entorhinal cortex (EC) than male 5xFAD mice treated with vehicle and female 5xFAD mice treated with TBR or vehicle (**B**). The 6-month-old male 5xFAD mice treated with TBR had significantly less amyloid β than either group of 5xFAD female mice (**C**). There were no between-group sex differences in amyloid beta in the 12-month-old mice (**D**–**F**). * *p* < 0.05. (∆, female mice; ●, male mice).” The correct legend appears below. Upon reanalysis, it was found that there was not a significant difference between male and female mice for the average amount of Aβ deposition. 

**Figure 11:**
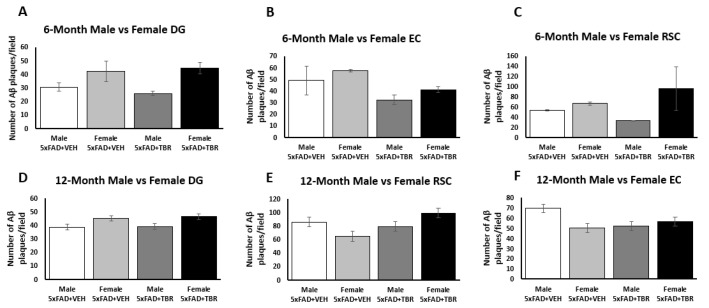
TBR did not reduce the number of amyloid β deposits in male 5xFAD mice compared to female (**A**–**F**).

### 2.4. Figure 12

In the original article, there were two duplicate images and an incorrect placement of a blot. For the 6-month-old 5xFAD cortex in the original article, the blot that was selected for Iba1 should have been set for the loading control GAPDH and the appropriate blot has replaced the top Iba1 blot in Figure 12. In Figure 12, the loading control for a 12-month-old hippocampus GAPDH was incorrectly chosen. We have removed this loading control and replaced it with the appropriate loading control blot. The group identifier was also incorrectly placed for this group and has been corrected. The authors have removed the incorrect images. The errors in the representative image selection did not impact the underlying analysis. The corrected Figure 12 appears below. 

## 3. Text Correction

### 3.1. In Abstract

There was an error in the original abstract stated that “TBR also protected against neuron loss, reduced activation of astrocytes and microglia, primarily in 6-month-old mice, and attenuated Aβ deposition.” Upon reanalysis, we found that TBR did not protect against neuron loss or attenuate Aβ deposition in treated 5xFAD mice. The original article has been updated. Corrected paragraph/abstract below.

**Abstract:** Combined treatments using polyphenols and omega fatty acids provide several therapeutic benefits for a variety of age-related disorders, including Alzheimer’s disease (AD). Previously, we found a commercial product, Total Body Rhythm (TBR), consisting of tart cherry extract, a potent polyphenol, and omega fatty acids, significantly reduced memory, and neuropathological deficits in the 192 IgG-saporin mouse model of AD. The present study assessed the efficacy of TBR for treating behavioral and neuropathological deficits in the 5xFAD model of AD. Both 6- and 12-month-old 5xFAD mice and age-matched wild-type controls received TBR (60 mg/kg) or the equivalent dose of vehicle (0.5% methylcellulose) via oral administration, every other day for two months. All mice were tested in the open field (OF), novel object recognition (NOR), and the Morris water maze (MWM) tasks. In addition, neuronal morphology, neurodegeneration, Aβ plaque load, and glial activation were assessed. TBR treatment reduced memory deficits in the MWM and NOR tests and lessened anxiety levels in the OF task, mostly in the 6-month-old male mice. TBR also protected and reduced activation of astrocytes and microglia, primarily in 6-month-old mice. These results suggest that the combination of tart cherry extract and omega fatty acids in TBR can reduce AD-like deficits in 5xFAD mice. 

### 3.2. In the Results Part

There was an error in the original article, in the first paragraph in *Section 3.5. Cresyl-Violet Imaging for Neuronal Morphology*, that stated: “a significant decrease in the number of pyknotic cells in 6-month-old 5xFAD mice treated with TBR”. Upon reanalysis, we found that TBR did not decrease the number of pyknotic neurons.

Corrected paragraph: Neuron morphology was assessed by staining sections with 0.1% cresyl-violet and counting the number of pyknotic neurons in the CA1 and CA3 areas of the hippocampus, as well as in the pyramidal layer 5 of the PFC (Figure 9A). Using a one-way ANOVA, it was revealed that there was a significant decrease in the number of pyknotic cells in 6-month-old 5xFAD mice treated with TBR.

There was an error in the original article, in the first paragraph in *Section 3.6. Amyloid β-Plaque Staining*, that stated: “A two-way independent *t*-test revealed a significant difference in the average number of Aβ plaque for the 6-month-old mice in the retro-splenial cortex between the four AD 6-month-old mice treated with TBR and the five AD mice treated with vehicle (Figure 10B). There was a significant difference in the average number of β-amyloid peptide between the five AD 12-month-old mice treated with TBR and the six AD mice treated with vehicle (Figure 10C). There were no significant differences in the dentate gyrus for either age group in the number of Aβ plaques. However, subsequent analyses indicated that this difference was due primarily to how the male mice responded to TBR treatment (Figure 11).” Upon reanalysis, we found that TBR did not protect against Aβ deposition. 

Corrected paragraph: A two-way independent t-test revealed there was no significant difference in the average number of Aβ plaque for the 6-month-old or 12-month-old mice (Figure 10B–G).

There was an error in the original article, in the first paragraph in *Section 3.7. Sex Differences in Number of Amyloid-β Plaques in 6-Month-Old Mice*, that stated: “One-way ANOVAs revealed significant sex differences in the average number of Aβ plaques for the 6-month-old 5xFAD mice. There was a significant difference in the number of Aβ plaques retrosplenial cortex (RSC) (*F*(3, 68) = 17.7579, *p* < 0.05), dentate gyrus (DG) (*F*(3, 72) = 10.4856, *p* < 0.05), and entorhinal area (EC) (*F*(3, 67) = 8.4006, *p* < 0.05) (Figure 11A–C). Using the Scheffè post hoc analysis, it was found that there was a significant difference in the number of Aβ plaques between the male 5xFAD mice treated with TBR and the male 5xFAD+VEH, female 5xFAD+VEH, and the female 5xFAD+TBR (*p* < 0.05). The 6-month-old female 5xFAD mice treated with TBR were not significantly different than the female mice treated with vehicle (Figure 11A,B). Using the Scheffè post hoc analysis, it was found that there was a significant difference between the male 5xFAD mice treated with TBR, female 5xFAD+VEH, and female 5xFAD+TBR (*p* < 0.05) in the DG. The 6-month-old female 5xFAD mice treated with TBR were not significantly different than the female mice treated with vehicle (Figure 11C).” Upon reanalysis, we found that TBR did not protect against sex-dependent Aβ deposition. 

Corrected paragraph: One-way ANOVAs revealed no significant sex differences in the average number of Aβ plaques for the 6-month-old or 12-month-old 5xFAD mice (Figure 11A–F).

### 3.3. In the Discussion Part

There was an error in the original Discussion section. In the first paragraph, it stated: “Our findings confirmed our hypotheses that mice receiving TBR would have fewer cognitive deficits, increased neuron protection, changes in glial cell functioning, and reduced β-amyloid load.” Upon reanalysis, we found that TBR did not protect against Aβ deposition. 

Corrected paragraph: The current study tested the combinatorial treatment of tart cherry extract and omega fatty acids (TBR) in 5xFAD AD mice at both 6 and 12 months of age. Our findings confirmed our hypotheses that mice receiving TBR would have fewer cognitive deficits and changes in glial cell functioning. Previous work from our lab has demonstrated that TBR can prevent loss of body weight in a cholinergic-toxin (192 IgG-saporin) mouse model of AD [22]. The finding that there were no significant differences between the 5xFAD animals and age-matched WT controls in weight or of activity in our study underscores the argument that the differences seen in the NOR and MWM tasks were due to disturbances in cognition rather than overall decrements in health or activity levels [28]. However, the findings of an increased level of anxiety in untreated 5xFAD mice may have influenced the outcome of the NOR tasks, although differences in the number of approaches to the objects were not observed, suggesting that the differences in the amount of time investigating the novel object were likely due to recognition memory differences rather than differences in anxiety levels or motivation.

In the fourth paragraph in the Discussion section, it stated: “In addition to cognitive sparing, we found that TBR was able to reduce the number of pyknotic cells in the hippocampus and prefrontal cortex of the mouse brain (Figure 9). Alzheimer’s disease, unlike normal aging, is associated with a large loss of neurons [35]. Furthermore, the total number of lost neurons is positively correlated with severity of symptoms [36]. Pyknotic cells are those which are undergoing necrosis or apoptosis. For both 6-and 12-month-old mice, there were reduced numbers of pyknotic cells in those animals that received TBR treatment. It is possible that TBR, through reducing oxidative stress and correction of fatty acid profiles was able to protect both neurons and glial cells.” Upon reanalysis, we found that TBR did not protect against neuron loss or attenuate Aβ deposition in treated 5xFAD mice.

Corrected paragraph: Although TBR reduced cognitive deficits, it was unable to reduce the number of pyknotic cells in the hippocampus and prefrontal cortex of the mouse brain (Figure 9). Alzheimer’s disease, unlike normal aging, is associated with a large loss of neurons [32]. Furthermore, the total number of lost neurons is positively correlated with the severity of symptoms [33]. Pyknotic cells are those which are undergoing necrosis or apoptosis. For both 6-and 12-month-old mice there was only an insignificant trend toward reduced numbers of pyknotic cells in those animals which received TBR treatment. Further research is needed to determine whether higher doses or newer formulations with higher amounts of omega-3 fatty acids might provide a neuroprotective effect in rodent models of AD.

In the sixth paragraph in the Discussion section, it stated: “We demonstrated that TBR was able to reduce amyloid beta levels within the retrosplenial cortex of the 6-month-old AD mice treated with TBR and the entorhinal cortex of 12-month-old mice.” Upon reanalysis, we found that TBR did not attenuate Aβ deposition in treated 5xFAD mice. 

Corrected paragraph: The 5xFAD mouse model is known to be an aggressive model, with rapid formation of amyloid plaques. TBR was not able to reduce amyloid beta levels within the 6-and 12-month-old AD mice, although a non-significant trend was observed in the retrosplenial cortex (RSC). The RSC constitutes a large portion of cortex in rodents, which corresponds to Broadman’s area 29 and 30 [37]. Although the RSC was identified by Broadman over 90 years ago, the structure and function of this region remain elusive. It is known that in sporadic AD there are volumetric differences in the RSC that are comparable with the atrophy seen within the hippocampus of humans [38]. It has been demonstrated that there are dramatic differences in size between species, and there is now a strong connection between the RSC cortex and a range of cognitive functions. According to an extensive review conducted by Vann and colleagues, the RSC has emerged as a key member of a network of brain regions, including the hippocampus and limbic system, and is involved in episodic memory, navigation, imagination, and planning for the future [37]. All of these functions are crucial for performing the memory tasks assessed in our study and could explain why we found such a significant difference at 48 h post-training in the MWM.

In the seventh paragraph in the Discussion section, it stated: “When exploring the impact that sex has on AD symptoms, we found a difference between the 6-month-old male and female 5xFAD mice on the NOR task and the number of amyloid β deposits. We found that male mice responded better to treatment on the NOR (Figure 6) than the female mice, in that they spent increased time exploring the novel object during testing.” Upon reanalysis, we found that TBR did not attenuate sex dependent Aβ deposition in treated 5xFAD mice.

Corrected paragraph: When exploring the impact that sex has on AD symptoms, we found a difference between the 6-month-old male and female 5xFAD mice on the NOR task. We found that male mice responded better to treatment on the NOR (Figure 6) than the female mice, in that they spent increased time exploring the novel object during testing. Understanding the relationship between amyloid β and cognitive impairment in AD is complex. Some argue that there is very little connection; indeed, patients can have varying degrees of cognitive deficits, even when having similar amyloid beta loads [39].

In the eighth paragraph in the Discussion section, it stated: “Our results show that TBR influences amyloid beta deposition in a sex-dependent fashion.” It also stated: “However, the degree to which amyloid β load is related to cognitive deficits is debatable, but our findings that males had reduced levels of amyloid β and responded more strongly to TBR therapy suggests that increased amyloid β may have contributed to the memory deficits observed in this study.” Upon reanalysis, we found that TBR did not attenuate sex-dependent Aβ deposition in treated 5xFAD mice. 

Corrected paragraph: Our results show that TBR does not influence amyloid beta deposition in a sex-dependent fashion. It has been demonstrated in multiple transgenic mice, including APPswe, PS1 double transgenic, and 3xTg mice, that female mice have increased amyloid β burden compared with male mice [40,41]. This increased burden of amyloid-β was also found to coincide with worse cognitive outcomes for female mice as well [41]. In addition, it has been demonstrated for over two decades that both macaque and human males respond preferentially to donepezil, one of the most common cholinesterase inhibitors [42,43]. Both polyphenols and omega fatty acids, through different mechanisms, have been shown to reduce amyloid β load. However, the degree to which amyloid β load is related to cognitive deficits is debatable and our results suggest that TBR can reduce cognitive deficits without significantly reducing amyloid beta levels.

In the ninth paragraph in the Discussion section, it stated: “Manipulation of sex hormone levels in the mice could be contributing to the sex differences observed in the behavioral and amyloid-β data of our study. It has been demonstrated that 5xFAD female mice can have increasing amyloid burden until 14 months of age, possibly due to influences of the Thy-1 promoter used to express the transgenes in this model of AD [11]. We hypothesize that either the tart cherry extract or the fatty acids influenced sex hormone differences occurring earlier in disease development, which then influenced outcomes; it is well known that within the human population and in animal models that females have higher rates of AD and that this difference cannot be attributed to simply living longer [47]. A recent comprehensive review using over 275 articles and meta-analysis found that women are more likely to suffer increased cognitive deterioration than men who are at the same AD disease stage [48]. Indeed, the same group who found a disparity between men and women in AD have found that episodic memory is a key factor in this discrepancy [49]. However, if the difference seen in the NOR in our study was primarily because of amyloid-β load, it could be argued that 12-month-old 5xFAD mice would be expected to have more amyloid-β than a 6-month-old female.” Upon reanalysis, we found that TBR did not attenuate sex dependent Aβ deposition in treated 5xFAD mice. 

Corrected paragraph: Manipulation of sex hormone levels in the mice could be contributing to the sex differences observed in the behavioral data of our study. It has been demonstrated that 5xFAD female mice can have an increasing amyloid burden until 14 months of age, possibly due to influences of the Thy-1 promoter used to express the transgenes in this model of AD [11]. We hypothesize that either the tart cherry extract or the fatty acids influenced sex hormone differences occurring earlier in disease development, which then influenced outcomes; it is well known that within the human population and in animal models that females have higher rates of AD and that this difference cannot be attributed to simply living longer [44]. A recent comprehensive review using over 275 articles and meta-analysis found that women are more likely to suffer increased cognitive deterioration than men who are at the same AD disease stage [45]. Indeed, the same group who found a disparity between men and women in AD have found that episodic memory is a key factor in this discrepancy [46].

### 3.4. In the Materials and Methods Part

There was an error in the original Section 2.4.1. Open-Field Testing, which stated: “The OF testing was used to assess spontaneous motor activity and anxiety [23,24]. Additionally, fecal boli counts were taken during each trial. Mice were tested in one 30 min trial at both pre- and post-treatment. For details, see [24].”

Corrected paragraph: The OF testing was used to assess spontaneous motor activity and anxiety [23]. Additionally, fecal boli counts were taken during each trial. Mice were tested in one 30 min trial at both pre- and post-treatment.

There was an error in the original Section 2.4.2. Novel Object Recognition, which stated: “The novel object recognition (NOR) task allows for the assessment of recognition memory in an environment with minimal stress [22,25–27]. We have published details of NOR procedure [24].”

Corrected sentence: The novel object recognition (NOR) task allows for the assessment of recognition memory in an environment with minimal stress [22,24,25].

There was an error in the original Section 2.4.3. Morris Water Maze (MWM), which stated: “The MWM task measures the ability to find a hidden platform in a pool of water and is used as an assessment of procedural learning and spatial memory [24,28,29].”

Corrected sentence: The MWM task measures the ability to find a hidden platform in a pool of water and is used as an assessment of procedural learning and spatial memory [26,27].

There was an error in the original Section 2.6.2. Amyloid β-Plaque Count, which stated: “For both 6- and 12-month-old mice, coronal sections were sampled from bregma at –1.28 mm and −2.92 mm. Sections were stained using curcumin, which labels plaques as efficiently as Aβ-specific antibodies [30]”.

Corrected sentence: For both 6- and 12-month-old mice, coronal sections were sampled from bregma at –1.28 mm and −2.92 mm. Sections were stained using curcumin.

## 4. In Reference Part

The original reference [24,27,30] should be deleted.

24. Maiti, P.; Bowers, Z.; Bourcier-Schultz, A.; Morse, J.; Dunbar, G.L. Preservation of dendritic spine morphology and postsynaptic signaling markers after treatment with solid lipid curcumin particles in the 5xFAD mouse model of Alzheimer’s amyloidosis. *Alzheimers Res. Ther.* **2021**, *13*, 37.
27. Paladugu, L.; Gharaibeh, A.; Kolli, N.; Learman, C.; Hall, T.C.; Li, L.; Rossignol, J.; Maiti, P.; Dunbar, G.L. Liraglutide Has Anti-Inflammatory and Anti-Amyloid Properties in Streptozotocin-Induced and 5xFAD Mouse Models of Alzheimer’s Disease. *Int. J. Mol. Sci.* **2021**, *22*.
30. Maiti, P.; Hall, T.C.; Paladugu, L.; Kolli, N.; Learman, C.; Rossignol, J.; Dunbar, G.L. A comparative study of dietary curcumin, nanocurcumin, and other classical amyloid-binding dyes for labeling and imaging of amyloid plaques in brain tissue of 5×- familial Alzheimer’s disease mice. *Histochem. Cell Biol.* **2016**, *146*, 609–625.

The authors apologize for any inconvenience caused. The original article has been updated.

## Figures and Tables

**Figure 12:**
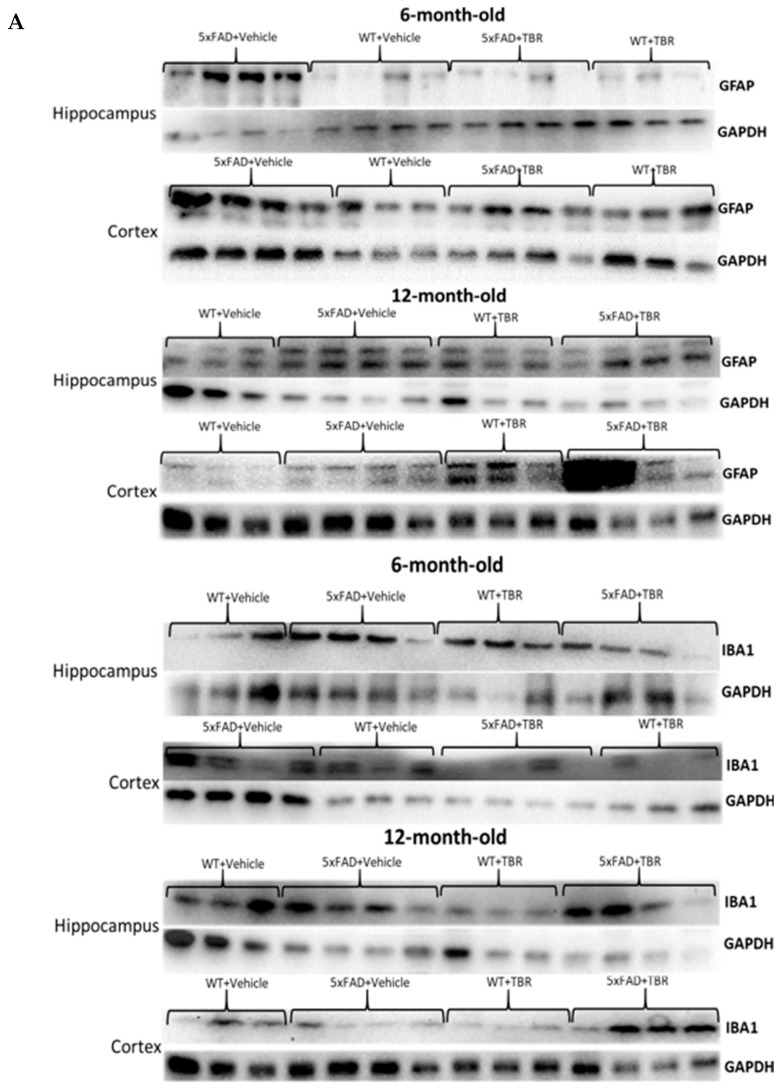

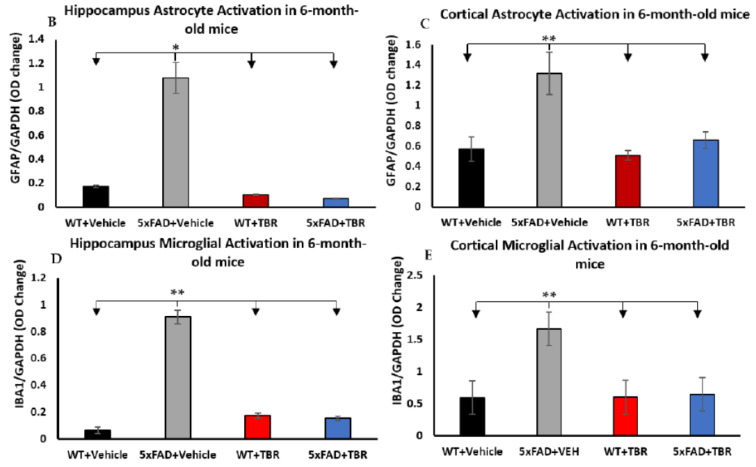
Western blots of GFAP and Iba1 levels in 6- and 12-month-old mice (**A**). There were significant between-group differences in hippocampal and cortical GFAP and Iba1 levels in the 6-month-old mice, but no significant between-group differences were observed in these measures for the 12-month-old mice (**B**–**E**). * *p* < 0.05; ** *p* < 0.05 for the 5xFAD mice, when compared with all other groups. Blots are representative images.

## References

[1] Bowers Z., Maiti P., Bourcier A., Morse J., Jenrow K., Rossignol J., Dunbar G.L. (2021). Tart Cherry Extract and Omega Fatty Acids Reduce Behavioral Deficits, Gliosis, and Amyloid-Beta Deposition in the 5xFAD Mouse Model of Alzheimer’s Disease. Brain Sci..

